# Fractal-based linear model of resting state hemodynamic response in fMRI

**DOI:** 10.1186/1471-2202-13-S1-P35

**Published:** 2012-07-16

**Authors:** Wonsang You, Sophie Achard, Jörg Stadler

**Affiliations:** 1 Special Lab Non-invasive Brain Imaging, Leibniz Institute for Neurobiology, Magdeburg, Germany; 2 GIPSA-lab, CNRS, UMR 5216, Grenoble, France

## 

Understanding endogenous dynamics of the brain has been one of crucial issues in neuroscience since it will disclose a huge default-mode functional network hidden behind resting state signals. Recent studies have shown that a resting state fMRI time series tend to exhibit the fractal property such as 1/f-type spectral density which is characterized by a fractal exponent [1]. There exist indirect evidences supporting that the fractal exponent is associated with neurophysiological activities [2], however the relevance of fractal behavior with functional connectivity has been still unclear.

Recently it was observed that the spontaneous fluctuation of cerebral blood volume also exhibits fractal properties [3]. Since fMRI is a non-invasive measurement of hemodynamic activities, it leads us to consider attributing the fractal behavior of BOLD signals to cerebral hemodynamics as well as neuronal activities. Motivated by this idea, we propose a fractal-based linear model of resting state hemodynamic response function (rs-HRF) whose behavior is summarized by a fractal exponent (See Figure 1). It comprises the infinite or large number of density functions with slowly decaying coefficients according to a fractal exponent while the classical HRF consists of just two density functions. We showed that the special condition of rs-HRF causes long memory in BOLD signals. The rs-HRF model also implies that a resting state BOLD signal can be well approximated as a fractionally integrated process (FIP) rather than the typical fractional Gaussian noise (FGN) [4].

**Figure 1:**
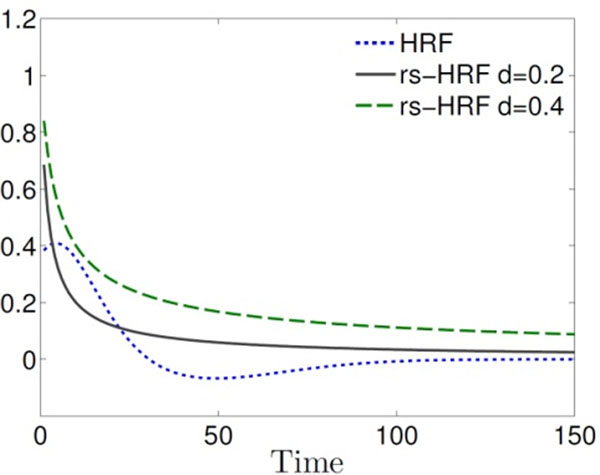
The plots of classical HRF (without delay) and rs-HRFs with two memory parameters. While the classical HRF may have undershoot, the rs-HRF has no undershoot but longer tail.

The simulation studies based on the rs-HRF model enables us to figure out the influence of hemodynamic fractal behavior on functional connectivity of resting state BOLD signals. First, we simulated a multivariate autoregressive process as an approximation of stationary resting state neuronal activity, and obtained its corresponding BOLD signals by convolving them with rs-HRF. Then, we observed the dissimilarity of wavelet correlation matrices between neuronal activities and BOLD signals on the basis of the symmetric Kullback-Leibler divergence which can be utilized as a measure of difference between two distributions. The dissimilarity of wavelet correlation increases in high frequency scales as the variance of fractal exponents increases while the dissimilarity approaches to zero in low frequency scales. These results suggest that the difference of fractal exponents between brain regions may cause apparent discrepancy of functional connectivity between neuronal activities and BOLD signals in high frequency scales.

## Conclusion

All of these results may give us insight into the dependence of functional connectivity on fractal behavior, and direct us to the default-mode functional network dominated by neuronal activities beyond fractal behavior of BOLD signals. While the linear relationship between neuronal activities and BOLD signals in evoked state has been well represented by HRF, it is questionable that the classical HRF is appropriate even for resting state since the model does not well reflect the fractal properties of cerebral hemodynamics as well as BOLD signals. In the future work, it will be valuable to constitute the nonlinear model of fractal behavior induced by cerebral hemodynamics as an extension of the classical Balloon model.
